# The Editor's Letter-Box

**Published:** 1919-05-24

**Authors:** Gladys M. E. Leigh


*To the Editor of* The Hospital.

Yours faithfully,

Geraldine Bremner.

22 Langham Street, Portland Place, W. 1, May 19, 1919.

*To the Editor of* The Hospital.

Sir,—The obvious reply to Miss Sheldon's question in the current number of The Hospital is: "Reform must first come from within." It is time the committees of the various trained nurses' institutions came to a definite and concerted resolution to revise not only the hours of work for their staffs but also their salaries.

In the country the sleeping difficulty seldom presents itself, in London it is the duty of the institutions to provide sleeping-quarters for their nurses, charging the usual fees paid by patients under these circumstances.

As to expensive railway fares, there are so many excellent co-operative societies now to be found in all large towns that patients in the surrounding districts can easily obtain *skilled* nursing without incurring the extra expense of fares for long journeys; moreover, patients who are in a position to pay the fees charged by London consultants have no excuse for the exploitation of the nursing profession. The alternative of being treated in a nursing home is always open to them, the fees range from five to sixteen guineas a week, exclusive of the use of the operating-theatre and additional bills for drugs and dressings.

If the public is given clearly to understand that the exploitation of the private nurse is no longer to be tolerated it will bring pressure to bear on the Government to an extent that will force the newly-created Ministry of Health to seriously consider the project of establishing paying hospitals to meet the needs of patients who are ineligible for admission into the wards of the voluntary hospitals and who are unable to pay private nursing and medical fees.

Surely never was there a more auspicious moment to launch a scheme which has been vaguely mooted for years; there are innumerable war hospitals, closed and closing, fitted with the most modern appliances, which could easily be adapted for civilian patients. Organised on a business footing, they would be self-supporting,

**The Editor's Letter-Box**—(*continued*).

and their inauguration would supply a national need, also institutions modelled on the lines I suggest would suffice to remedy two evils: (1) The exploitation of the private nurse. (2) The exploitation of the public by the owners of private nursing homes.

Yours faithfully,
Gladys M. E. Leigh.

